# Classification of Osteonecrosis of the Femoral Head Stage on Radiographic Images Using Deep Learning Techniques

**DOI:** 10.3390/bioengineering12121319

**Published:** 2025-12-03

**Authors:** Hyun Hee Lee, Joeun Jeong, Taehoon Shin, Dong-Sik Chae

**Affiliations:** 1 Department of Orthopedic Surgery, International St. Mary’s Hospital, Catholic Kwandong University College of Medicine, Incheon 22711, Republic of Korea; emprany@ish.ac.kr; 2 Department of Mechanical and Biomedical Engineering, Ewha Womans University, Seoul 03760, Republic of Korea; jje5976@ewhain.net (J.J.); shinage@gmail.com (T.S.); 3 Graduate Program in Smart Factory, Ewha Womans University, Seoul 03760, Republic of Korea

**Keywords:** ONFH, deep learning, normative modeling, variational autoencoder

## Abstract

While magnetic resonance imaging (MRI) is effective for detecting early-stage osteonecrosis of the femoral head (ONFH), it is often expensive and less accessible; conversely, radiography is more widely accessible but has limited sensitivity for early-stage diagnosis. We developed a deep learning approach using radiographic images to effectively classify ONFH stages, providing a more accessible method for early diagnosis and disease stage differentiation. The dataset consisted of 909 hip radiographs, yielding 1818 femoral head images (grade 0:1495; grade 1:80; grade 2:114; grade 3:93; grade 4:36). A U-Net model was used to segment the femoral heads, achieving a Dice similarity coefficient (DSC) of 0.977 on the test set, allowing precise localization of the region of interest. A variational autoencoder (VAE) was then trained using 1270 grade-0 images for training and 112 for validation to construct a normative latent distribution representing healthy femoral heads. When ONFH data from all grades were projected into the latent space, significant differences in Mahalanobis distance distributions were observed across most ONFH stages. No significant difference was found between grades 0 and 1 (*p* = 0.06), consistent with known radiographic subtlety. However, grades 2–4 showed significant deviation from grade 0, and significant differences were also observed among mid- and late-stage grades. These findings demonstrate that the proposed method effectively separates healthy and diseased femoral heads and captures gradewise structural progression within the latent space. This radiograph-based normative modeling approach offers an accessible alternative to MRI for ONFH stage differentiation, particularly in resource-limited clinical environments. Although early-stage differentiation remains challenging, the results highlight the potential of radiograph-based deep learning systems to improve diagnostic efficiency and support future automated ONFH staging workflows.

## 1. Introduction

Osteonecrosis of the femoral head (ONFH) is a pathological condition characterized by necrosis of the bone tissue in the femoral head due to an interruption in its blood supply. According to the internationally recognized Association Research Circulation Osseous staging system, ONFH is classified into five stages based on symptom severity. Grade 0 represents a healthy condition, whereas grades 1–4 correspond to an increasing severity of symptoms. The pre-collapse stage includes grades 1 and 2, in which femoral head collapse has not yet occurred [1,2]. Conversely, the post-collapse stage encompasses grades 3 and 4, in which structural collapse of the femoral head is evident.

Treatment strategies are tailored to the disease stage, with early intervention being crucial for slowing the progression of bone necrosis. Therefore, rapid diagnosis is essential to ensure timely management and minimize the long-term impact of ONFH on joint function and patient quality of life. The importance of early detection cannot be overstated, as it directly influences therapeutic outcomes and preserves the structural integrity of the femoral head [3].

Various imaging modalities, including radiography and magnetic resonance imaging (MRI), have been employed in the diagnosis of ONFH. MRI is the gold standard for diagnosing ONFH [4,5,6]. It offers the substantial advantage of distinguishing between healthy conditions and ONFH in grade 1. However, MRI has limitations such as higher costs and reduced accessibility compared with radiography. In contrast, radiography is affordable and widely accessible, making it the first-line diagnostic tool for patients with hip pain. However, its sensitivity in the early stages of ONFH, particularly grade 1, is limited because radiographic abnormalities are either absent or minimal. This limitation poses a challenge to early diagnosis.

Despite these drawbacks, radiography remains a critical diagnostic tool because of its practicality and accessibility. If the ability to differentiate ONFH stages using radiographic imaging could be improved, it would enhance the overall efficiency of ONFH diagnosis. Early-stage identification via radiographic imaging could facilitate timely interventions and better outcomes for patients with ONFH. Previous radiograph-based classification studies have consistently reported poor performance in distinguishing grade 1 from normal conditions due to minimal radiographic changes. Despite the practicality of radiographic imaging in diagnosing ONFH, previous studies have highlighted considerable challenges in differentiating the disease stages, particularly grade 1. In a study by Chee et al. [7], classification experiments using radiographic images demonstrated poor performance in distinguishing grade 1 from other stages. Similarly, Li et al. [8] concluded that grade 1 was indistinguishable from healthy conditions and combined it with normal data, treating it as non-diseased. These findings underscore the inherent limitations of radiographs in early detection and highlight the critical need for computational methods capable of extracting subtle structural variations not easily perceived by human observers.

To address these limitations, our study introduces a radiograph-based deep learning framework that uses MRI-confirmed ARCO grading as ground truth. This design leverages the accessibility of radiographs while anchoring disease labels to MRI, thereby overcoming the ambiguity of early-stage radiographic findings. Furthermore, by integrating automated femoral head segmentation and normative modeling, our approach focuses the analysis on anatomically relevant regions and quantifies latent structural deviation across ONFH stages [9].

We developed a deep learning approach using radiographic images to effectively classify ONFH stages, providing a more accessible method for early diagnosis and disease stage differentiation [9]. The main contributions of this study are as follows: (1) we introduce a radiograph-based deep learning framework that leverages MRI-confirmed ARCO grading to overcome the limitations of early-stage ONFH detection using radiographs; (2) we propose a two-stage pipeline combining automated femoral head segmentation and VAE-based normative modeling to characterize gradewise structural deviations; (3) we demonstrate that the latent-space deviation metrics derived from the normative model provide statistically significant separation for most ONFH grades; and (4) we provide a fully automated, clinically accessible workflow that may support triage, early screening, and future automated staging systems in resource-limited environments.

## 2. Materials and Methods

### 2.1. Methodology

**Figure 1:**
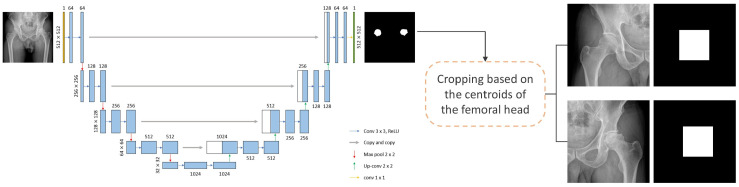
Architecture of the U-Net model.

This study follows a structured methodology that consists of four primary stages: (1) dataset construction and MRI-confirmed ARCO labeling, (2) automated femoral head segmentation using a U-Net-based model, (3) VAE-based normative modeling to characterize gradewise latent-space deviations, and (4) quantitative evaluation using Mahalanobis distance distributions and statistical validation. A schematic pipeline is presented in Figure 1 to illustrate the full workflow from raw radiographs to latent-space analysis. Each stage of the methodology is described in detail in the subsequent subsections to enhance transparency and reproducibility.

This study utilized a total of 909 hip radiographs obtained from a single-center institution, yielding 1818 femoral head images after separating the left and right sides. The distribution of femoral heads across ONFH grades was as follows: grade 0 (*n* = 1495), grade 1 (*n* = 80), grade 2 (*n* = 114), grade 3 (*n* = 93), and grade 4 (*n* = 36), as summarized in Table 1. For the segmentation task, 220 radiographs were annotated and divided into training, validation, and test sets consisting of 176 (80%), 22 (10%), and 22 (10%) images, respectively. For the VAE-based normative modeling, 1270 healthy femoral head images were used for training and 112 for validation, while gradewise deviation analysis was performed using an independent set of 255 grade 0 images and all available images from grades 1–4.

Because this dataset is derived from a single-center clinical archive and includes patient imaging protected under IRB regulations, it cannot be publicly released. However, the dataset can be made available upon reasonable request to the corresponding author, in accordance with institutional data-sharing policies.

### 2.2. Study Design, Data Collection, and Labeling

The dataset used in the present study presents a severe class imbalance, which is a common characteristic of medical data, where normal data typically dominate. This imbalance leads to the classification models being biased toward normal data during training, resulting in inadequate learning of the characteristics of diseased grades and poor classification performance. To address these challenges, this study employed normative modeling [10], leveraging the abundance of normal data. This approach allows for the analysis of disease heterogeneity and the assessment of substantial differences not only between grades 0 and 1 but also among other grades.

To mitigate class imbalance, we applied class-balanced loss weighting, oversampling of minority ONFH grades, and extensive data augmentation during model development.

The study was divided into two main stages:

1. Segmentation of the Femoral Head Region

Using the widely adopted U-Net architecture [11], the femoral head region, where the key features of ONFH manifest, was segmented on the radiographic images. This step ensured the precise localization of the area of interest and facilitated downstream analysis [12].

2. Normative Modeling with Variational Autoencoder (VAE)

A VAE [13] was trained exclusively on the femoral head data from healthy (grade 0) cases. The differences among the grades were examined by analyzing the latent space of the trained VAE. This approach highlighted the variations between grades 0 and 1, as well as other grades, enabling a deeper understanding of ONFH progression and inter-grade distinctions [14,15].

This methodology not only mitigated the challenges posed by class imbalance but also provided a robust framework for analyzing disease-specific characteristics in ONFH using radiographic imaging.

This study included 909 hip radiographs obtained from a single-center institution. The dataset consisted of hip radiographs in the anteroposterior view, and all data were from individuals aged 20 years or older. For ONFH patient data, radiographic and magnetic resonance images were required to ensure accurate annotation. Patients with hip osteoarthritis, pathological fractures, or history of internal fixation in the hip region were excluded.

The grade labels for ONFH were determined on the basis of expert radiological evaluations. For accurate labeling, MRI examinations performed within ±30 days of the radiographs were required, and ARCO grading was determined independently by two fellowship-trained hip surgeons with consensus resolution for disagreements. Each radiographic image included the left and right femoral heads, resulting in 1818 femoral head images. The distribution of femoral head images across the ONFH grades is shown in Table 1.

To prevent model bias and improve generalizability, the dataset was split into training, validation, and test sets with strict separation by patient, and cross-validation was used during model development. Overfitting was further mitigated through early stopping, weight decay, dropout, and monitoring validation loss across training epochs. This rigorous data collection and labeling process ensured the reliability and consistency of the dataset, enabling robust analysis and modeling of ONFH stages.

### 2.3. Femoral Head Segmentation Model

On hip radiographs, the femoral head, where the key symptomatic features of ONFH manifest, occupies a localized region compared with the overall image size. Background information outside the femoral head region can act as noise, potentially hindering an effective analysis. To address this, a segmentation model was trained to isolate the femoral head region from radiographic images.

The segmentation model employed was U-Net, which is a widely used architecture for medical image segmentation. The structure of the U-Net model is illustrated in Figure 1; it features a standard configuration with four downsampling and four upsampling operations. This design effectively captures the local and global features of the femoral head region, ensuring accurate segmentation. A total of 220 hip radiographic images were segmentation labeled using ITK-SNAP [16] and partitioned into training, validation, and test sets consisting of 176, 22, and 22 images, respectively. Data augmentation techniques, such as random horizontal and vertical flips, were implemented during model training. To improve robustness and reduce overfitting, we applied random flips, intensity augmentation, and contrast variation during segmentation training. Although more advanced CNN encoder backbones such as ResNet or DenseNet could theoretically be incorporated, integrating these architectures into the current segmentation pipeline was not feasible due to constraints in dataset size, annotation volume, and the fixed-resolution cropping workflow [17,18,19,20,21]. Instead, to enhance robustness within the scope of this study, we included comparative evaluation using commonly used baseline architectures such as classical U-Net and DeepLabV3, allowing performance contextualization without requiring substantial structural modification to the pipeline [22].

The segmentation masks generated from the trained U-Net were used to identify the centroid coordinates of the right and left femoral heads. Based on these coordinates, 512 × 512 images tightly encompassing the femoral head were cropped from the original radiographic images. Then, the cropped image was used as the input for the subsequent model for disease grading (Section 2.4).

### 2.4. Normative Modeling

**Figure 2:**
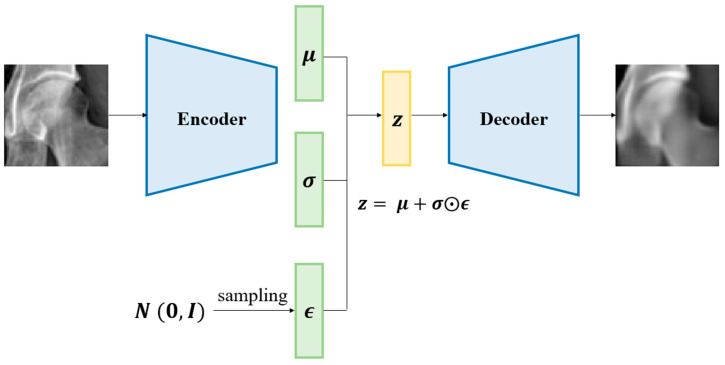
Architecture of the variational autoencoder.

Normative modeling is a deep learning approach designed to analyze the heterogeneity of diseases effectively [23]. This method enables the assessment of disease severity by quantifying how each patient deviates from the normative pattern defined by the distribution of healthy controls [3,7]. A VAE was employed as the framework for normative modeling (Figure 2) [8].

The VAE extracts meaningful features from the input data, represents them as a latent vector z through an encoder, and recovers the original input data from the latent vector through a decoder. During the encoding process of the VAE, latent vector z was forced to follow a Gaussian distribution [5,24]. In normative modeling, the VAE is trained on data from only healthy controls so the resultant Gaussian distribution serves as the reference for healthy controls. The Gaussian reference distribution was constructed using the latent representations of MRI-confirmed grade 0 femoral heads, ensuring consistency between radiographic inputs and MRI-based ground truth labels. During inference, patient data are transformed by the trained VAE into latent vectors that deviate from the reference Gaussian distribution to varying degrees, depending on the disease stage. Mahalanobis distance (MD) was computed between each latent vector and the multivariate Gaussian distribution of the grade 0 controls to quantify the degree of deviation, and MD was used as a continuous deviation metric rather than a categorical classifier to capture subtle structural variations [25,26,27]. MD was chosen because it provides an interpretable, covariance-aware metric of deviation from the normative distribution, making it particularly well suited for normative modeling frameworks. The threshold for defining abnormal deviation was determined using validation-set receiver operating characteristic (ROC) analysis, with the optimal cutoff selected using the Youden index to ensure an objective and data-driven boundary for abnormality in the latent space. This strategy enables differentiation of ONFH grades using a model trained with only healthy participant data and may provide insights into the heterogeneity of ONFH across different stages in the latent space.

A total of 1270 healthy femoral head images were used for training and 112 for validation, and early stopping was applied if the validation loss failed to improve for 50 epochs to avoid overfitting. After training was completed, ONFH data from other grades were inputted into the trained VAE to evaluate the deviation of the resulting latent vectors from the Gaussian distribution formed by the grade 0 data. The dataset for the gradewise distribution analysis comprised 255, 80, 114, 93, and 36 images of ONFH grades 0, 1, 2, 3, and 4, respectively. During inference, the Mahalanobis distance was used to quantify the differences between the latent vectors for grade 0 and those for other grades. The Welch t-test was used to statistically verify the significance of the differences in the Mahalanobis distance distributions across grades. This approach allowed a robust evaluation of gradewise distinctions in the latent space and provided statistical evidence for the separation of ONFH grades based on their latent vector characteristics.

### 2.5. Experimental Environment and Details

The experiments were conducted using PyTorch and four NVIDIA RTX A6000 Ada GPUs. The training and evaluation details for the femoral head segmentation model and normative modeling via VAE included the femoral head segmentation model (batch size, 16; epochs, 200; loss function, dice loss; optimizer, AdamW; learning rate, 0.0001; and evaluation metric, dice similarity coefficient (DSC) [Dice loss and DSC were used to focus the training on accurate segmentation of the femoral head region.]) and normative modeling via VAE (batch size, 256; epochs, a maximum of 1000 epochs [early stopping was applied if the validation loss did not improve for 50 consecutive epochs]; loss function, weighted sum of binary cross-entropy loss [BCE loss] and Kullback–Leibler divergence loss [KL loss] [28] with a warm-up weight for KL loss in the range [0.0001–0.01]; optimizer, Adam; learning rate, 0.0005; and latent vector size, 64). Regularization methods including dropout and weight decay were incorporated to further reduce overfitting risk in both the segmentation and VAE models.

The loss function of the VAE model incorporated a combination of BCE loss to ensure accurate reconstruction and KL divergence to regularize the latent space. A warm-up schedule for the KL weight was employed to stabilize training. The model used early stopping to prevent overfitting and reduce computational overhead. These carefully selected configurations ensured that the models were optimized for their respective tasks, providing reliable and robust results for segmentation and normative modeling. All experiments were conducted using Python 3.10, PyTorch 2.1.0, CUDA 12.1, and torchvision 0.16.0. Supporting libraries included NumPy 1.26, SciPy 1.11, scikit-learn 1.3, and OpenCV 4.8. These version specifications are provided to ensure reproducibility of the computational environment.

## 3. Results

### 3.1. Femoral Head Segmentation

The performance of the femoral head segmentation model was evaluated using the DSC. The model achieved its best validation at epoch 164 with DSC of 0.974 and demonstrated a DSC of 0.977 on the test dataset, indicating an excellent segmentation performance. To contextualize the achieved segmentation accuracy, we additionally compared the U-Net model with commonly used baseline architectures such as classical U-Net and DeepLabV3. The proposed model demonstrated superior performance (baseline DSC range: 0.951–0.967), confirming the robustness of the adopted segmentation framework.

**Figure 3:**
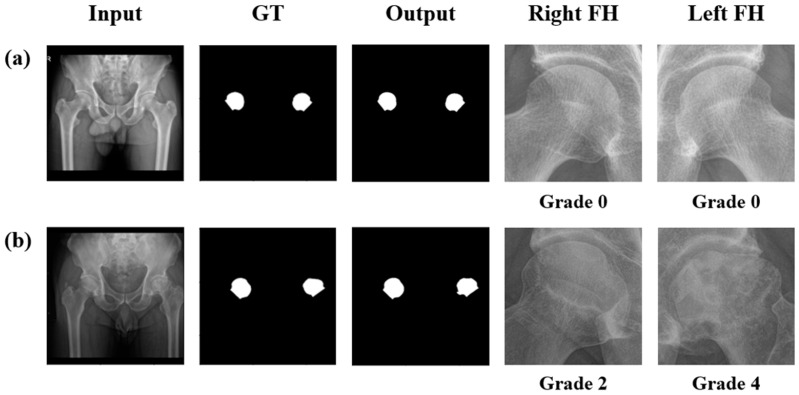
Input radiograph, ground-truth (GT) segmentation mask, output mask, and cropped right and left femoral head (FH) images of two example cases. The first case involves FHs classified as grade 0 (**a**), and the second case involves a right FH classified as grade 2 and a left as grade 4 (**b**).

Figure 3 illustrates the segmentation performance of the U-Net model on the test images for the healthy and diseased cases. The predicted masks closely matched the ground-truth masks, demonstrating that the segmentation model effectively learned to identify femoral head regions with high accuracy. The model accurately segmented the femoral head regions even in cases involving varying degrees of femoral head collapse due to ONFH. These findings validated the robustness of the segmentation model for healthy and diseased femoral heads. The image patches tightly surrounding the segmented femoral heads were cropped (Figure 3) and fed into a VAE-based normative model. All these steps were performed in a fully automated end-to-end manner.

### 3.2. Normative Modeling of ONFH

**Figure 4:**
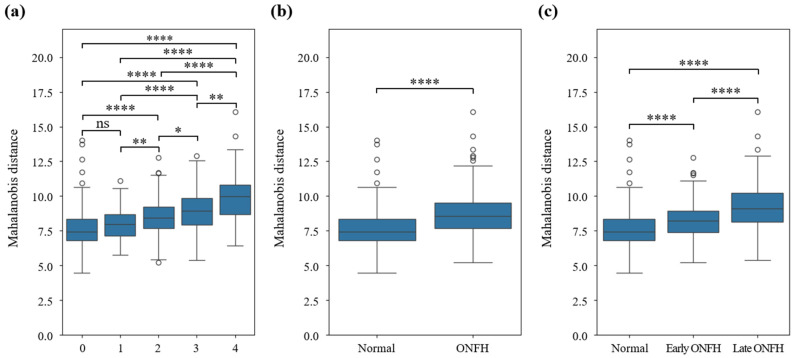
Welch t-test results for each grade (0–4). (**a**) Healthy and ONFH groups; (**b**) healthy, early-stage ONFH (pre-collapse); and (**c**) late-stage ONFH (post-collapse) groups. Not significant (ns): 0.05 < *p* ≤ 1, *: 0.01 < *p* ≤ 0.05, **: 0.001 < *p* ≤ 0.01, ****: *p* ≤ 0.0001. ONFH, osteonecrosis of the femoral head.

Figure 4 displays the statistics of the Mahalanobis distance from the Gaussian distribution of the healthy control data for the test radiographic data for all ONFH grades. According to the Welch t-test used to assess the difference in the Mahalanobis distance across ONFH grades, the following observations were made. Welch’s *t*-test, pre-specified in the study design to handle unequal variances and sample imbalance across grades, demonstrated statistically significant differences among most grade pairs, supporting the robustness of latent-space separation achieved by the proposed normative model.

Consistent with previous findings that grade 1 is difficult to visually distinguish from healthy conditions, no significant difference was observed in the Mahalanobis distance distribution between grades 0 and 1 (*p* = 0.06). Regarding, other grade comparisons, significant differences were observed in the Mahalanobis distance distributions between any pair of ONFH grades, including grade 0 versus (vs.) grades 2–4, grade 1 vs. grades 2–4, and grades 2, 3, and 4. These statistical findings provide quantitative evidence for the validity of the deviation-based analysis and reinforce that the model captures meaningful gradewise structural differences.

A significant difference was observed between the healthy group (grade 0) and ONFH disease group (defined as a union of grades 1–4), demonstrating that the VAE latent representation effectively separated the healthy and diseased femoral heads. Significant differences were observed when comparing healthy vs. pre-collapse (grades 1–2), healthy vs. post-collapse (grades 3–4), and pre-collapse vs. post-collapse stages.

## 4. Discussion

This study investigated a novel approach for diagnosing and staging ONFH using radiographic imaging combined with deep learning techniques. The methodology integrated femoral head segmentation and normative modeling to address the challenges associated with the early-stage diagnosis and heterogeneity of ONFH. The findings demonstrate the capability of the model to capture structural changes in ONFH progression. Additionally, the results validate the utility of the VAE-based normative modeling approach for analyzing ONFH heterogeneity and identifying significant latent space differences across disease stages.

The femoral head segmentation model based on U-Net demonstrated excellent performance. The ability of the model to accurately segment normal and diseased femoral heads, including those with varying degrees of collapse, highlights its robustness and practical utility in capturing regions critical for diagnosing and staging ONFH. This precise segmentation is essential for removing irrelevant background information and ensuring that the model focuses on localized pathological features of ONFH. The comparison with baseline segmentation architectures further confirmed that the proposed U-Net configuration provided more accurate anatomical localization, which is essential for reliable downstream analysis. A limitation of the current study is that segmentation performance was evaluated using DSC alone. Jaccard (IoU), a complementary overlap metric, was not computed because the segmentation pipeline and evaluation codebase were originally optimized around DSC. Future work will incorporate IoU-based evaluation to provide a more comprehensive assessment of segmentation accuracy. While deeper CNN encoder designs such as ResNet and DenseNet may potentially enhance feature extraction, incorporating these architectures was not practical within the current study due to dataset scale, annotation availability, and pipeline compatibility. Therefore, instead of restructuring the entire segmentation framework, we adopted a feasible alternative by benchmarking against widely used baseline models (classical U-Net and DeepLabV3), which provided a meaningful robustness assessment within the constraints of our data and computational resources.

The application of normative modeling using a VAE provided valuable insights into the latent space representation of the ONFH grades. Significant differences in Mahalanobis distance distributions were observed between most ONFH grades, demonstrating the potential of this approach for capturing disease-specific characteristics. However, no significant differences were found between grades 0 and 1. This aligns with findings in existing literature, indicating the difficulty in visually distinguishing these stages using radiographs. The significant separations observed between later grades and between the healthy and diseased groups underscore the ability of this method to differentiate the structural changes associated with disease progression. Recent cutting-edge ONFH studies have predominantly focused on MRI-based deep learning classification, radiomics-derived biomarkers, or CNN-based radiographic grading [9]. While these approaches have demonstrated promising diagnostic utility, they generally rely on MRI availability or supervised classification frameworks that require balanced datasets across all disease stages. In contrast, our radiograph-based normative modeling strategy introduces a fundamentally different perspective by quantifying latent-space deviation relative to MRI-confirmed healthy controls. This deviation-based approach aligns with recent trends in normative modeling and representation learning while offering superior accessibility in clinical environments where MRI is limited. Thus, our framework complements and extends current cutting-edge research by providing a scalable, radiograph-driven method for analyzing gradewise ONFH progression. The significance testing using Welch’s t-test strengthened the interpretability of latent-space deviations, confirming that the observed differences were not due to sampling imbalance but reflected true gradewise structural variation.

Despite these promising results, this study highlights areas for further investigation. First, the use of the Mahalanobis distance as a metric for grade differentiation is effective but may not fully capture subtle variations between the early stages. Future research should explore alternative evaluation methods and incorporate additional features or models to enhance grade-specific distinctions. Second, the dataset exhibited a significant class imbalance, with normal femoral heads dominating the data. Normative modeling effectively leverages this imbalance, and expanding the dataset to include more diseased samples, particularly for grades 1 and 4, enhances the robustness of the model. Future studies may incorporate additional statistical validation frameworks or multi-metric deviation measures to enhance the robustness of gradewise differentiation [29].

Lastly, the goal of developing an automated ONFH staging model remains a critical direction for future research. Such a model could integrate the findings of this study to improve its diagnostic accuracy and efficiency in clinical settings, particularly in resource-limited environments where MRI access is constrained. By combining segmentation, latent space analysis, and classification, future systems can provide comprehensive support for ONFH diagnosis and treatment planning.

This study demonstrated the feasibility and potential of using radiographic imaging with deep learning-based normative modeling to classify ONFH stages. These results provide a foundation for further exploration and refinement and contribute to the development of accessible and efficient diagnostic tools for ONFH.

This study has several limitations that should be acknowledged. First, the dataset exhibited substantial class imbalance, particularly with fewer samples in grades 1 and 4, which may limit the sensitivity of latent-space separation for early-stage disease. Second, because the dataset was sourced from a single-center institutional archive, the generalizability of the model to multi-center or heterogeneous populations remains to be validated [30,31]. Third, segmentation evaluation relied solely on DSC, as the pipeline was not configured to compute Jaccard (IoU), which will be incorporated in future studies. Fourth, although comparisons with baseline architectures such as classical U-Net and DeepLabV3 were included, a broader comparison with deeper CNN backbones (e.g., ResNet- or DenseNet-based models) could not be performed due to dataset and pipeline constraints. Finally, the normative model quantifies latent deviation but does not provide direct stage classification, which will require future extension toward a fully automated clinical staging system.

## 5. Conclusions

Femoral head segmentation experiments demonstrated that the model accurately predicted not only healthy femoral heads but also those affected by ONFH, even in advanced stages. Using segmented data for normative modeling, the results revealed no significant differences in the Mahalanobis distance distributions between grades 0 and 1. However, significant differences were observed among all the other grade pairs, highlighting the ability of the model to capture key variations in ONFH progression.

Importantly, the normative model successfully separated healthy vs. diseased femoral heads and distinguished pre-collapse (grades 1–2) vs. post-collapse (grades 3–4) stages with statistical significance, demonstrating its potential as a radiograph-based tool for early triage and disease stratification in environments where MRI access is limited.

Future research should explore alternative evaluation methods that better capture grade-specific differences beyond Mahalanobis distance. The goal was to develop a model capable of automatically classifying ONFH grades by leveraging the findings of this study, and these advances have considerably enhanced the efficiency and accuracy of ONFH diagnosis and staging in clinical practice.

## Figures and Tables

**Table 1:** Distribution of femoral head images by ONFH grade.

	Grade				
	0	1	2	3	4
Number of femoral heads	1495	80	114	93	36

ONFH, osteonecrosis of the femoral head.

## Data Availability

The data presented in this study are available upon reasonable request from the corresponding author. The data are not publicly available due to institutional data protection policies.

## Abbreviations

The following abbreviations are used in this manuscript:

ONFH	Osteonecrosis of the femoral head
MRI	Magnetic resonance imaging
VAE	Variational autoencoder
DSC	Dice similarity coefficient
BCE loss	Binary cross-entropy loss
KL loss	Kullback–Leibler divergence loss
Vs.	Versus
